# Prolonged exposure to acid and bile induces chromosome abnormalities that precede malignant transformation of benign Barrett’s epithelium

**DOI:** 10.1186/1755-8166-5-43

**Published:** 2012-11-29

**Authors:** Manisha Bajpai, Hana Aviv, Kiron M Das

**Affiliations:** 1 Division of Gastroenterology and Hepatology, Department of Medicine, UMDNJ-Robert Wood Johnson Medical School, 1 Robert Wood Johnson Place, New Brunswick, NJ, 08903, USA; 2 Department of Pathology, UMDNJ-Robert Wood Johnson Medical School, 1 Robert Wood Johnson Place, New Brunswick, NJ, 08903, USA

**Keywords:** Barrett’s epithelium, BAR-T, Barrett’s epithelium carcinogenesis model, Aneuploidy, Polyploidy, Chromosomal aberrations, Genetic instability

## Abstract

**Abstract:**

Barrett’s esophagus (BE) is an asymptomatic, pre-malignant condition of the esophagus that can progress to esophageal adenocarcinoma (EAC). BE arises typically in individuals with long-standing gastroesophageal reflux disease (GERD). The neoplastic progression of BE has been extensively studied histologically and defined as a metaplasia- dyplasia- carcinoma sequence. However the genetic basis of this process is poorly understood. It is conceived that preclinical models of BE may facilitate discovery of molecular markers due to ease of longitudinal sampling. Clinical markers to stratify the patients at higher risk are vital to institute appropriate therapeutic intervention since EAC has very poor prognosis. We developed a dynamic in-vitro BE carcinogenesis (BEC) model by exposing naïve Barrett’s epithelium cell line (BAR-T) to acid and bile at pH4 (B4), 5min/day for a year. The BEC model acquired malignant characteristics after chronic repeated exposure to B4 similar to the sequential progression of BE to EAC in vivo.

**Aim:**

To study cytogenetic changes during progressive transformation in the BEC model.

**Results:**

We observed that the BAR-T cells progressively acquired several chromosomal abnormalities in the BEC model. Evidence of chromosomal loss (-Y) rearrangements [t(10;16) and dup (11q)] and clonal selection appeared during the early stages of the BEC model. Clonal selection resulted in a stabilized monoclonal population of cells that had a changed morphology and formed colony in soft agar. BAR-T cells grown in parallel without any exposure did not show any of these abnormalities.

**Conclusions:**

Prolonged acid and bile exposure induced chromosomal aberrations and clonal selection in benign BAR-T cells. Since aneuploidy preceded morphological/dysplastic changes in the BEC model, chromosomal aberrations may be an early predictor of BE progression. The [t(10;16) and dup(11q)] aberrations identified in this study harbor several genes associated with cancer and may be responsible for neoplastic behavior of cells. After further validation, in-vivo, they may be clinically useful for diagnosis of BE, progressing to dysplasia/esophageal adenocarcinoma.

## Background

Barrett’s esophagus (BE) is a specialized columnar intestinal metaplasia containing goblet cells that replaces the native esophageal squamous mucosa in individuals with long-standing gastroesophageal reflux disease (GERD). BE is a pre-malignant condition of the esophagus that can progress to esophageal adenocarcinoma (EAC) with poor prognosis [1]. Patients with histological BE are 30–125 times more susceptible to developing EAC compared to those without BE. The neoplastic progression of BE has been extensively studied and defined as a metaplasia- dysplasia- carcinoma sequence. However the genetic basis of BE pathogenesis is poorly understood.

Progressive genetic instability and clonal selection has been proposed as possible basis of neoplastic evolution in BE [2]. Alterations in *TP53* and *P16* genes, aneuploidy and loss of heterozygosity (LOH) have been identified as characteristic early events of clonal evolution in the molecular pathogenesis of BE [2-7]. Only 0.5-1% of BE patients progress to EAC annually [8], hence large cohorts have to be followed over several years to obtain statistically relevant data [2,9]. Therefore a preclinical model of BE would facilitate longitudinal sampling to follow development and progression of neoplasia from non neoplastic epithelium [10].

Rodent and canine BE models utilized surgical anastomosis to induce chronic reflux of gastric acid and/or duodenal contents, including bile into the esophagus, to induce metaplasia and dysplasia and EAC [11,12]. Several ex-vivo and cell line models of BE enabled understanding of the possible contributory role of acid and bile to the molecular mechanism(s) of BE pathogenesis [13-17]. 

The dynamic in-vitro model of BEC is developed from exposing naïve benign BAR-T cells to acid (pH4) and bile glycochenodeoxycolic acid (GCDA) for 5 mins a day, for about year [14]. Induction of double strand DNA breaks after acute acid and bile acids exposure have been strongly suggested in BAR-T cells [11,12]. BAR-T cells are hTERT immortalized Barrett’s epithelium cell line [13]. BAR-T cells in the BE Carcinogenesis(BEC) model acquired malignant characteristics in a sequential progression and changed from benign to neoplastic epithelium. Although the BAR-T cells spontaneously lost *CDKN2A* during the initial passages yet the cell cycle checkpoints were intact [13] during the initiation of the BEC model. We observed loss of *TP53* gene expression after 45 weeks of exposure of acid and bile [14] in the BEC model accompanied by changes in cell morphology, loss of contact inhibition (foci formation), loss of adherence dependence (colony formation in soft agar) and finally tumor formation in nude mice [14]. To our knowledge this is the only sequential dynamic in-vitro model that shows BE progression to neoplasia a direct consequence of acid and bile exposure, the noxious components of gastroesophageal refluxate contributing to clinical pathogenesis of BE.

Based on comprehensive cytogenetic analysis of 150 cell lines and tumor cells both in vitro and in vivo a new concept on the pathways of karyotypic evolution of cells in culture was put forward by Mamaeva in 1998 [15]. The report unveiled that cells in culture qualitatively display karyotypic variability corresponding to two distinct stages- establishment stage, and stabilization stage in the evolution of the cell line. During the establishment stage massive changes in the numerical and structural rearrangement of chromosomes occur resulting in heterogeneity of clones. Duration of this stage is ruled by the time necessary for selection of dominant and stable clones. A stabilized cell line has minimal karyotype heterogeneity and a clearly defined modal class of chromosome numbers [15].

Our study demonstrates for the first time divergence of “karyotype evolution” in BAR-T cell line due to selection pressure in the presence of prolonged intermittent acid and bile exposure. The karyotype evolution may be a result of genetic instability and heterogeneity [15,16] yet acid and bile treatment appears to facilitate selection of cell clones most adapted for existence in-vitro in adverse environment. This novel report also demonstrates chromosomal loss and rearrangements (duplication and translocation) as a direct consequence of chronic acid and bile exposure. It is clearly evident that the BAR-T cells in an environment (acid and bile, pH4, B4) conducive of disease progression undergo clonal divergence resulting from chromosomal aberrations. Eventually, clonal selection leads to monoclonal cell population with aneuploid karyotypes that have changed morphology, form soft agar colonies and tumor in nude mice. However, in the absence of environmental factors the karyotype evolution in untreated BAR-T leads to stabilized polyploid clones that retain Y-chromosome and do not form tumor or colonies on soft agar.

## Results

### BAR-T cells accumulate multiple chromosomal aberrations upon prolonged intermittent B4 exposure

**Figure 1:**
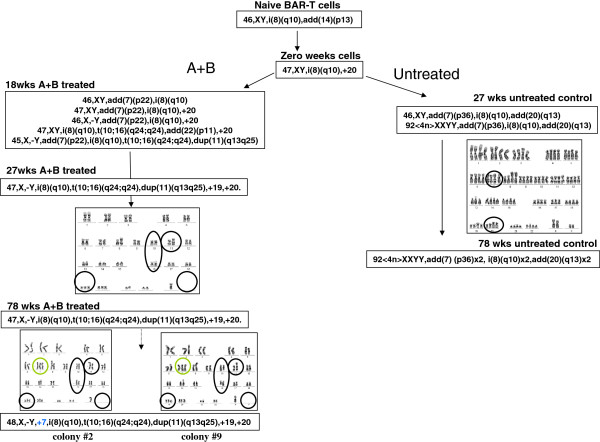
Karyotype analyses of BAR-T cells exposed to acid and bile for different time points.

The BEC model was initiated with naïve BAR-T cells that had the karyotype 46,XY,i(8)(q10) [13]. After 18 wks of chronic intermittent (5min/day) B4 exposure, BAR-T cells displayed 5 clonal variants based on different karyotypes: 46,XY,add (7) (p22), i(8) (q10)/47,XY, add (7) (p22), i(8) (q10),+20/46,X,-Y, add (7) (p22), i(8) (q10), +20/47,XY, i(8) (q10), t(10;16) (q24;q24), add (22) (p11), +20/45,X,-Y, add (7) (p22), i(8) (q10), t(10;16) (q24;q24), dup(11) (q13q25) (Figure 1 and Table 1). The 18 wks BAR-T cells growing in parallel without any acid or bile exposure did not display these characteristics (Figure 1). Later, at 27 weeks of the BEC model the B4 exposed BAR-T cells displayed monoclonal karyotype. This karyotype 47,X,-Y,i(8)(q10),t(10;16)(q24;q24),dup(11)(q13q25),+19,+20 was different from the naïve cells and resembled a combination of variant clones observed at 18 wks of the BEC model.

**Table 1:** Changes in cytogenetic profile of BAR-T cell line due to chronic intermittent acid and bile (pH4) exposure

	**Chr #**	**(−Y)**	**add(7p)**	**i(8)(q10)**	**t(10;16)**	**dup(11q)**	**(+19)**	**(+20)**
BAR-T A+B 18 wks	46,47	(+) 2/14	(+) 12/14	(+)	(+) 2/14	(+) 1/14		(+) 7/14
BAR-T A+B 27 wks	47	(+)		(+)	(+)	(+)	(+)	(+)
BAR-T A+B 48 wks	47	(+)		(+)	(+)	(+)	(+)	(+)
BAR-T A+B 65 wks	47	(+)		(+)	(+)	(+)	(+)	(+)
BAR-T A+B 78 wks	47	(+)		(+)	(+)	(+)	(+)	(+)

The BAR-T cells in the BEC model acquired several chromosomal abnormalities like- loss of Y chromosome, a translocation between the long arms of chromosomes 10 and 16 [t(10;16)(q24;q24)], duplication of the long arm of chromosome 11, dup(11) (q13q25) and trisomies 19 and 20 (Table 1) but maintained the isochromosome 8q of the naïve cells. This karyotype signature appeared to be stable since it was maintained in all subsequent time points examined in the BEC model up to 78wks. Since the untreated cells growing in parallel did not display these abnormalities, the chromosomal changes observed in BEC cells appear to be a consequence of prolonged acid and bile induced DNA damage.

### The transformed BAR-T cells in the BEC model are monoclonal

About 11% of 78 wks B4 treated BAR-T cells formed colonies in soft agar (i.e. 110 out of the 1000 cells plated per well). Each colony growing in soft agar may be representative of an independently transformed clone. To examine this hypothesis, nine random distinctly separated colonies were successfully picked from 78 weeks B4 treated BAR-T cells growing in soft agar plate. All of these 9 colonies (clones #1- 9) were stabilized and subjected to cytogenetic analysis. Each of the 9 colonies not exhibited striking similarity in karyotype very similar to the parent 78wks B4 cells (Table 2). Additionally, these clones had an added characteristic, they had trisomy 7 not found in parent 78wks B4 cells: 48,X,-Y,+7,i(8)(q10),t(10;16)(q24;q24),dup(11)(q13q25),+19,+20 (Table 2). It may be possible that trisomy 7 was essential for soft agar colony formation.

**Table 2:** Cytogenetic profile of cell lines derived from colonies on soft agar arising from78 wks A+B treated, BAR-T cells

	**Chr #**	**(−Y)**	**(+7)**	**i(8)(q10)**	**t(10;16)**	**dup(11q)**	**(+19)**	**(+20)**
BAR-T A+B 78 wks	47	(+)		(+)	(+)	(+)	(+)	(+)
Colony 1 cell line	48	(+)	(+)	(+)	(+)	(+)	(+)	(+)
Colony 2 cell line	48	(+)	(+)	(+)	(+)	(+)	(+)	(+)
Colony 3 cell line	48	(+)	(+)	(+)	(+)	(+)	(+)	(+)
Colony 4 cell line	48	(+)	(+)	(+)	(+)	(+)	(+)	(+)
Colony 5 cell line	48	(+)	(+)	(+)	(+)	(+)	(+)	(+)
Colony 6 cell line	48	(+)	(+)	(+)	(+)	(+)	(+)	(+)
Colony 7 cell line	48	(+)	(+)	(+)	(+)	(+)	(+)	(+)
Colony 8 cell line	48	(+)	(+)	(+)	(+)	(+)	(+)	(+)
Colony 9 cell line	48	(+)	(+)	(+)	(+)	(+)	(+)	(+)

### The BAR-T cell line spontaneously develops polyploidy after prolonged culture

BAR-T cells grown in parallel as untreated controls displayed karyotype similar to the naïve cells. However a mixture of 46(2n) and 92(4n) chromosomes bearing clones with add (7p) and add (20q) chromosome segments not found in naïve cells (Table 3 and Figure 1) appeared at 27 weeks. Much later (at 76wks) all cells are uniformly 4n with 92 chromosomes. It is notable that even at 76 wks these control BAR-T cells retain the Y-chromosome, which is lost in the BEC model as early as 18 weeks of A+B exposure. The add (7p) and add (20q) chromosome segments and development of polyploidy do not transform the cells since they are unable to form colonies in soft agar and may therefore be considered non-neoplastic.

**Table 3:** Cytogenetic profile of untreated BAR-T cells during prolonged culture

	**Chr#**	**-Y**	**add(7p)**	**i(8)(q10)**	**t(10;16)**	**dup(11q)**	**+19**	**+20**	**add(20q)**
BAR-T 0 wks	46			+					
BAR-T 27 wks	46		+	+					+
	92		+	+					+
BAR-T 76 wks	91		+	+					+

### Test for authenticity of BAR-T cell line during development of the BEC model

STR (short tandem repeat) analysis was performed to rule out possible cross contamination between cell lines. We performed this analysis on naïve and 78 weeks B4 exposed BAR-T cells. All the 10 identifiers used for STR analysis were common for both groups of cells. This confirmed that cells in the BEC model were authentic and derived from the parent BAR-T cell line. It may be worth mention that the BAR-T cell line was derived from a male patient and had both X and Y chromosome in the naïve cells as well as the untreated BAR-T cells. Therefore it is notable that Amelogenin Y was not detected in the transformed (78wks, B4) BAR-T cells. This further confirmed the loss of Y chromosome during transformation in these cells (Table 4).

**Table 4:** STR (number of repeats at each locus) profile of BAR-T cell line

**Markers**	**78wks(A+B)**	**naive BAR-T**
D5S818	16	16
D21S11	24	24
D16S539	9, 12	9, 12
D5S818	7	7
CSF1PO	14	14
D8S1179	13	13
D5S818	16	16
D21S11	24	24
FGA	21, 24	21, 24
AMELOGENIN	X	X, Y

## Discussion

Chronic exposure of cells to oxidative stress results in increased genomic instability [17,18] characterized by numerical (aneuploidy or polyploidy) or structural chromosomal alterations (such as breaks, fusion, translocation, deletion, duplication etc.). Several hypotheses support contribution of chromosomal aberrations toward the development of malignancies [19]. The combination of genetic instability and clonal expansion have been implicated in progression of BE to EAC [9].

The BEC model displays development of genomic instability and clonal selection/expansion during karyotype evolution that is characteristically different from the untreated BAR-T cell line growing in parallel. It may be mentioned that the BEC model also displays morphological and neoplastic changes not observed in the paired untreated cells [14].

Most established tumor cell lines exhibit karyotype evolution during long term culture [15,16]. The initial establishment stage of these cell lines is marked by karyotypic heterogeneity caused by genomic instability. Clones most adapted to growth conditions are selected as the cell line reaches stabilization stage with minimum karyotype heterogeneity [15]. We could not find any mention of karyotypic evolution in cell lines after hTERT immortalisation in literature. Therefore, the changes, as observed in the untreated hTERT immortalized benign BAR-T cells during prolonged continued in-vitro culture, is a unique observation. More intriguing is the finding is that “physiological” agents such as acid and bile modulate karyotype evolution by influencing clonal variation and clonal selection in the BEC model.

The loss of chromosome Y observed in the BEC model is common in several types of human cancers including prostate carcinoma, renal cell carcinoma, acute promyelocytic leukemia, and head and neck squamous carcinoma [20-22]. Most cells derived from biopsy specimens of BE patients exhibit loss of the Y chromosome [23]. This chromosomal and phenotypic abnormalities have been suggested to be characteristic of the metaplasia-dysplasia-carcinoma sequence of BE pathogenesis [24].

Trisomy 7 has been reported in a wide variety of tumors of mainly epithelial origin, but also in some mesenchymal and neurogenic neoplasms [25]. It was also detected in the non-neoplastic regions in the vicinity of these tumors [26]. Trisomy 7 with concurrent increased expression of *epidermal growth factor receptor (EGFR) gene* located on this chromosome and *increased EGF binding* was observed in biopsies of Barrett’s epithelium [27]. Elevated levels of the *EGFR* has been identified as a common component of multiple cancer types and appear to promote solid tumor growth [28]. Therefore Trisomy 7, which was observed in the cell lines derived from soft agar colonies possibly, exacerbates their tumorogenic potential since only 11% of 78 wks cells of the BEC model expressed this abnormality.

Amplification of the chromosome llq13 region are frequently found in carcinomas of the breast and of the head and neck region. In these carcinomas, amplification of the 11q13 region might serve as a prognostic marker that identifies a subgroup of patients at increased risk. INT2-FGFR3, HSR1-FGF4 and CCND1 (Cyclin D1), a gene that regulates the G1/S transition of the cell cycle are some of the syntenic genes co-amplified in the 11q13 region [29]. The amplification is usually low (3 to 10 copies), and physically linked to chromosome 11. Squamous cell carcinomas of the esophagus with chromosome 11q13 amplification indicated simultaneous CCND1 gene amplification [30]. Amplification of the region 11q23 simultaneously with the *proto-oncogene MLL (myeloid/lymphoid leukemia)* is a characteristic development in acute myeloid leukemia [31]. Thus the duplication of the long arm of chromosome 11 found in our cell line may represent a low level of amplification of these and several other tumor promoting genes located on the chromosome 11 (complete list can be viewed at http://www.ornl.gov/sci/techresources/Human_Genome/posters/chromosome/chromo11.shtml). Duplication of a region on the long arm of chromosome 11 may be the first step in the transformation process.

DNA content increased (4n) by clearly delineated genome doubling in the untreated BAR-T cells. This may be endopolyploidy that arises from variations of the canonical G1–S–G2–M cell cycle that replicate the genome without cell division [32]. Endoreplication is common in cancer cells and is considered as a precursor to aneuploidy that leads to oncogenesis [33]. Polyploidy, (4n population) has been correlated with premalignant epithelium in EAC as a predictor of progression [7,34-36]. Limited studies suggest that genome instability increases with age in mammals [37-39]. Therefore, it is possible that 4n BAR-T cells display a typical ageing phenomenon [40]. It is unknown if development of polyploidy *is common in hTERT immortalized cell lines* after prolonged culture (1yr) and provides any survival advantage to the cells. Lack of cancer-specific gene alterations or lack of proper selection pressure may explain why tumorigenicity was not achieved in the untreated BAR-T cells [14].

This study reinforces the clinical fact that karyotype changes are detectable before the appearance of dysplasia (changes in cell morphology) in BE [9]. Several acquired genetic abnormalities, such as gene mutation, gene deletion, loss of heterozygosity, aberrant methylation, aberrant gene expression, and chromosomal aberrations, have been proposed as markers for diagnosis of BE progression [24,36,41-44]. However, translocation between long arms of chromosome 10 and 16, t(10;16)(q24;q24) is a novel finding from this study. Genes located on the long arm of chromosomes 10 and 16 are involved in myriad of cancer conditions are summarized in Table 5 (source: http://atlasgeneticsoncology.org/). It is possible that some of these genes may have been disrupted as a consequence of unknown breakpoints causing (t10;16) and giving an evolutionary advantage to the cells. It however remains to be established if this is a unique event in the transformed BAR-T cells or a frequent early event in the progression of BE to adenocarcinoma using patients’ BE tissue.

**Table 5:** Genes implicated in causing cancer located on the chromosome fragments 10q24 and 16q24 (reviewed and summarized from source: http://atlasgeneticsoncology.org/)

**Gene name**	**Location**	**Cancer connection**
*BTRC (beta-transducin repeat containing)*	10 q24	Several mutations of the gene have been reported in prostrate [45], breast [46] and gastric cancers [47]. Overexpression of the protein was detected in melanomas [48], hepatoblastomas [49] and colorectal [50], pancreatic [51] and breast carcinomas [52].
*LOXL4 (lysyl oxidase-like 4)*		*LOXL4 mRNA* was expressed in MDA-MB-231 highly invasive breast cancer cells, but not in poorly invasive and non-metastatic breast cancer cells MCF7 and T47D [53]. LOXL4 was over-expressed in most invasive HNSSC primary or metastatic tumors and cell lines, primary tumors of oral cavity as well as thyroid gland whereas no expression was detected in normal epithelial cells [54].
*NFkappa B2* nuclear factor of kappa light polypeptide gene enhancer in B-cells 2 (p49/p100)		rearrangement of *NFkappa b2 gene locus* has been found in many forms of lymphomas [55].
*PAX2 (Paired box gene 2)*		It has been proposed as a useful marker of prostate cancer as well as predictor of severity of kidney cancers [56]
*PDCD4 (Programmed Cell Death 4)*		Expression attenuated with progression in human tumors of the lung, colon, prostate and breast; diagnostic and prognostic for colon cancer staging with decreased expression in adenomas and a further decrease in stage 1 adenocarcinomas.
*CBFA2T3 (core-binding factor*, runt domain, alpha subunit 2 translocated to 3)	Chromosome 16 q24	Loss of normal *function of CBFA2T3 may* be a key event in the early stage of breast cancer [57]. LOH on the whole 16q22-qter region is frequently detected in breast and prostate cancer [58].
*CDT1 (chromatin licensing* and DNA replication factor 1)		*CDT1 is a potential oncogene*, highly expressed in cancer cell lines CaSki, HeLa, LNcap, MCF7, MDAMB231, and Saos.
*FBXO31 (F-box protein 31)*		Tumor suppressor down-regulated in breast cancer cell lines relative to normal breast expression and cause G1 phase cell cycle arrest of the MDA-MB-468 cell line. This region is frequently deleted in several human cancers causing loss of heterozygosity [59].

## Conclusions

We observed that prolonged acid and bile exposure induced chromosomal aberrations and clonal selection in benign Barrett’s epithelial cells (BAR-T) and lead to development of neoplasia in the BEC model. Absence of proper selection pressure may explain why the untreated cells growing in parallel showed distinctly different karyotype evolution and remained benign. Chromosomal changes in the BEC preceded morphological/dysplastic changes reported earlier [14]. Therefore chromosomal aberrations may be early predictors of BE progression. Most of chromosomal aberrations identified in this study are associated with cancer and may be responsible for neoplastic progression in BEC model. Yet two unique events observed in the BEC model: dup11q and t(10;16) deserve further validation, in-vivo. They may be clinically useful for diagnosis of BE, progressing to dysplasia/esophageal adenocarcinoma.

## Methods

### Cell culture

The BAR-T cell line was treated with acid and bile, glycochenodeoxycholic acid at pH4(B4) for 5 minutes everyday for more than 65 weeks to develop the BEC model. Untreated cells grown in parallel served as controls. Cells were collected and frozen in liquid nitrogen every 8–10 weeks from both treated and untreated groups for karyotyping. Nine independent colonies derived from 78 weeks B4 treated BAR-T cells were picked from soft agar and cultured independently into 24 well plates. Each of these 9 clones was expanded and frozen.

### Cytogenetic analysis

Cytogenetic analyses were performed with cells at different time points starting with naïve cells up to 78 weeks of B4 treated cells at about 10 weeks intervals (Figure 1). BAR-T cells at metaphase, PD~150, are obtained by colcemid arrest and hypotonic treatment with pre-warmed 0.075M KCI, fixed and washed in freshly made Carnoy’s (3:1 absolute methanol: glacial acetic acid), dropped onto precleaned microscope slides and air dried. Trypsin G-banding is performed following a modification of Seabright’s method [60]. Cytogenetic analysis was also performed on each of the 9 colonies that developed in soft agar derived from 78 weeks B4 treated BAR-T cells. Cells from 78 weeks B4 treated cells and parallel untreated BAR-T cells from different times were also analyzed.

### STR analysis

BAR-T cells from early passage and 78 weeks after B4 treatment were used for small tandem repeat (STR) analysis to rule out contamination of the cell line during prolonged cell culture. Genomic DNA was extracted by the phenol-chloroform-isoamyl alcohol method and 0.5 ng was used for amplification following the AmpFlSTR® Profiler™ PCR Amplification kit instructions. Amplified samples were analyzed by injecting into a capillary on the ABI PRISM® 310 Genetic Analyzer. GeneScan® software automatically analyzed the collected data, which was then imported into Genotyper® software for automatic genotyping of alleles.

## Competing interests

The authors declare that they have no competing interests.

## Authors’ contributions

MB – Made substantial contributions to conception and design and acquisition of data. Dr. Bajpai was involved in drafting the manuscript and revising it critically for important intellectual content. HA – performed karyotype analysis and interpreted the data. KMD – was involved in the inception of the project and drafting the manuscript and critically revised it for important intellectual content and gave the final approval of the version to be submitted to the Molecular Cytogenetics journal. All authors read and approved the final manuscript.
